# Operation Blanks

**Published:** 1891-03

**Authors:** 


OPERATION BLANKS. Containing directions for the preparation of: I. The Patient; II. The Room and the Bed; III. Dressings, etc.; IV. Medicines, etc., to which is added a list of Instruments required in various operations. By W. W. KEEN, M. D., Professor of Surgery in the Jefferson Medical College, Philadelphia. Published by Lea Brothers & Co., 706–708 Sansom Street, Philadelphia, Pa.

In the haste incident many times to the preparation for an operation, some of the essentials are liable to be overlooked, and these blanks will serve as a reminder to the busy surgeon. We commend them as a useful part of the nurse's outfit also.

As a surgeon of world-wide repute, and as Professor of Surgery in the Jefferson Medical College, of Philadelphia, Dr. Keen has had great experience in the requirements for operations of every description. The present blanks are elaborated from similar forms used by him for many years, and are a part of his system of preparations as detailed in his paper on The Organization of an Operation, in the *American Journal of Medical Sciences* for January, 1891. They will be found a great convenience by surgeons engaged in every line of operative work. The Operation Blanks are arranged for checking directions to be given to nurses, and for securing from dealers all the drugs and materials which may be necessary. The List of Instruments is classified according to the nature of the operation, and ensures that nothing shall be forgotten. The blanks are bound in blocks containing fifty and carrying a permanent list of instruments. Each block, therefore, answers for fifty operations. The price is fifty cents.

